# Isolation and Characterization of a Biosurfactant Producing Strain *Planococcus* sp. XW-1 from the Cold Marine Environment

**DOI:** 10.3390/ijerph19020782

**Published:** 2022-01-11

**Authors:** Ping Guo, Weiwei Xu, Shi Tang, Binxia Cao, Danna Wei, Manxia Zhang, Jianguo Lin, Wei Li

**Affiliations:** College of Environmental Science and Engineering, Dalian Maritime University, Dalian 116026, China; guoping214@dlmu.edu.cn (P.G.); weiwei7017@126.com (W.X.); tangshi1875@dlmu.edu.cn (S.T.); chaobin@dlmu.edu.cn (B.C.); dlmuwdn@126.com (D.W.); zmx@dlmu.edu.cn (M.Z.)

**Keywords:** petroleum-degrading bacteria, biosurfactant, low temperature, bioremedation

## Abstract

One cold-adapted strain, named *Planococcus* sp. XW-1, was isolated from the Yellow Sea. The strain can produce biosurfactant with petroleum as sole source of carbon at low temperature (4 °C). The biosurfactant was identified as glycolipid-type biosurfactant species by thin-layer chromatography (TLC) and Fourier transform infrared spectroscopy (FTIR). It reduced the surface tension of water to 26.8 mN/m with a critical micelle concentration measurement of 60 mg/L. The produced biosurfactant possesses high surface activity at wide ranges of temperature (−18–105 °C), pH values (2–12), and salt concentrations (1–18%). The biosurfactant exhibited higher surface activity and higher growth rate of cells with hexadecane and diesel as carbon source. The strain *Planococcus* sp. XW-1 was also effective in degrading crude oil, after 21 days of growth at 4 °C in medium with 1% crude oil and 1% (*v*/*v*) bacteria broth, 54% of crude oil was degraded. The results suggest that *Planococcus* sp. XW-1 is a promising candidate for use in the bioremediation of petroleum-contaminated seawater in the Yellow Sea during winter. This study reported for the first time that *Planococcus* isolated from the Yellow Sea can produce biosurfactant using petroleum as the sole carbon source at low temperature (4 °C), showing its ecological role in the remediation of marine petroleum pollution.

## 1. Introduction

Offshore oil exploration, oil tanker crashes, oil pipeline leaks, oil waste water discharge, and subsea oil leakage have led to a large amount of oil entering the ocean [1]. The impact of oil entering the ocean is huge. It will affect the growth of marine life, destroy the marine ecological environment, reduce the value of the coastal environment, destroy coastal facilities, and reduce the self-purification capacity of the ocean [2,3]. Since low temperature will affect the migration and transformation process of oil and reduce bioavailability, oil spills that occur at low temperature environments are often more harmful [4].

Microbial remediation is based on the metabolic activities of microorganisms, and the damaged ecological environment is repaired by the degradation and transformation of toxic and harmful substances. Microbial remediation has characteristics which make it low cost, good effect, and environmentally friendly [5]. However, the oil degradation effect of petroleum-degrading bacteria will be affected by the ambient temperature. Low temperature will affect the physical and chemical properties of petroleum pollutants [6]. At low temperatures, the evaporation rate of volatile components is delayed, the petroleum hydrocarbon components of long-chain alkanes are insoluble or form solids, and the bioavailability is significantly reduced [7,8]. Some psychrophilic bacteria can promote the emulsification and dissolution of petroleum hydrocarbons by producing biosurfactants, thereby increasing degradation of petroleum hydrocarbons [2,9]. Biosurfactant production by organisms such as *Pseudomonas* spp., *Acinetobacter* spp., *Bacillus* spp., *Streptomyces* spp., *Rhodococcus* spp., *Achromobacter* spp., *Brevibacterium* spp., and *Arthobacter* spp. have been well studied [2,10]. However, research on biosurfactant production by *Planococcus* spp. is scanty. Normally, members of this genus are Gram positive and able to grow at low temperatures and high salt concentrations [11,12]. The genus *Planococcus* was reported generally for hydrocarbon degradation in comparison with biosurfactant secretion [13]. *Planococcus maitriensis* Anita I was isolated, hich was the first time physical properties of biosurfacant from *Planococcus* appeared in the literature [13,14]. 

Large-scale biosurfactant production from *Planococcus* was reported in 2014 [13,15]. Biosurfactants have functions such as dispersing, solubilizing, emulsifying, foaming, penetrating, wetting, etc., which are characterized by low impact on the environment, low surface tension and interfacial tension, wide temperature adaptation range, and salt resistance [16,17]. Therefore, it is widely used in the fields of medicine, agriculture, food, environmental restoration, and oil extraction [18,19]. In situ remediation of petroleum pollution by adding surfactant-producing strains can improve the remediation effect and reduce the cost of remediation. Research on the bacteria with the abilities in surfactant-producing and petroleum-degrading are mainly focused on mesophiles, and less research is focused on bacteria screened from the cold marine environment. *Halomonas* sp. ANT-3b, screened from Antarctica, can emulsify n-hexadecane at a low temperature [20]. Some *Rhodococcus* have also been found to produce surfactant at low temperatures [21,22]. Giuduce and other scholars have screened a number of surfactant-producing strains from Antarctica, mainly including *Rhodococcus*, *Pseudomonas*, and *Sphingomonas* [23,24,25,26,27]. Therefore, the petroleum-degrading bacteria that produce surfactants at low temperature should be studied in depth. 

The surface seawater temperature range of the Yellow Sea is very low in winter (about 1~13 °C) [28]. In cold seasons, several oil spills have occurred in this sea area [29]. One of the main difficulties in bioremediation of petroleum pollution in the low temperature environment is the lack of cold-adapted bacteria with the abilities in surfactant producing and petroleum degrading. However, most of these bacteria have been isolated from the Polar and sub-Polar regions, and few are from China. Since indigenous bacteria are more adapted to local environments than non-native species, it is best to use autochthonous bacteria for oil spill bioremediation. So, the goal of this work was to isolate efficient cold-adapted surfactant-producing petroleum-degrading bacteria from the north Yellow Sea and analyze the characteristics of the biosurfactant produced. 

## 2. Materials and Methods

### 2.1. Culture Medium 

Experiments were conducted using 2216E liquid medium, 2216E agar medium, and man-made marine culture (MMC) inorganic salt liquid medium [30].The 2216E media were purchased from Qingdao Haibo Biotechnology Co., Ltd. (Haibo Biotechnology Co., Ltd, Qingdao, China). The 2216E liquid medium consisted of (g/L): peptone, 5; yeast extract, 1; and high-phosphoric acid, 0.1. The 2216E agar medium was composed of (g/L): peptone, 5; yeast extract, 1; high-phosphoric acid, 0.1; and agar, 15. The MMC liquid medium contained (g/L): NaCl, 24; KH_2_PO_4_, 2.0; Na_2_HPO_4_, 3.0; NH_4_NO_3_, 1.0; MgSO_4_•7H_2_O, 7.0; KCl, 0.7; and trace elements. During preparation of the media, the pH was adjusted to 7.4, followed by autoclaving at 121 °C for 20 min. The trace elements contained (mg/L): CaCl_2_, 0.02; CuSO_4_, 0.005; FeCl_3_•6H_2_O, 0.5; ZnSO_4_•7H_2_O, 0.1; and MnCl_2_•4H_2_O, 0.005.

### 2.2. Isolation of the Bacteria

#### 2.2.1. Isolation of the Petroleum Degrading Bacteria

The surface seawater samples were collected in winter from the Dalian Port Cruise Terminal (38°56′7.30″ N, 121°39′34.37″ E), which located on the west coast of the north Yellow Sea. The temperature, salinity, and pH of the seawater are 3.4 °C, 3.2%, and 8.12, respectively. 

5 mL sea water were added to 100 mL of sterile MMC. Crude oil (1% *v*/*v*) was added as the sole source of carbon and energy and cultures were incubated at 4 °C with agitation at 180 rpm for 20 days. 5 mL of cultures were then used to inoculate the next round of culturing by addition to sterilized MMC containing 1 mL of crude oil. The cultures were again grown for 20 days at 180 rpm, and this process was repeated three times. The cultures with petroleum hydrocarbon biodegradation ability were transferred to the 2216E plates with the dilution rates of 10^−4^, 10^−5^, 10^−6^, 10^−7^, and 10^−8^, respectively. The single colony was separated by a streak plate method for three times. Pure strains were stored on nutrient agar slant at 4 °C in the refrigerator. Abilities of the isolated strains in biosurfactant production and diesel oil biodegradation were tested for further selection [31].

#### 2.2.2. Surface Tension Measurement

Surface tension measurement was performed with the procedure in previous literatures [32,33]. The isolates were cultured in conical flasks (250 mL capacity) containing 100 mL of MMC. Each conical flask was inoculated with isolated bacteria (grown 24 h in 2216E liquid medium at 4 °C and 180 rpm to attain an initial cell concentration OD_600_ = 1.5). Diesel oil was used as the sole carbon source and the cultures were grown at 4 °C and 180 rpm for 10 days. The culture was centrifuged at 6000× *g* for 20 min to remove cells, and then 20 mL of the cell-free supernatant was transferred to a petri dish to measure its surface tension by using a Wilhelmy plate-type tensiometer (BZY-101, Fangrui instrument Co., LTD, Shanghai, China). The surface tension measurement was carried out at room temperature after dipping the platinum plate in the cell-free supernatant for a while in order to attain equilibrium conditions. The measurement was repeated three times and an average value was obtained.

#### 2.2.3. Diesel Oil Degradation Measurement

Diesel oil degradation was measured as described by Xue et al. [34]. Cell-free supernatant was extracted thrice using a total volume of 60 mL of petroleum ether and the organic phase was filtered by anhydrous sodium sulfate to obtain the rest of crude oil. Residual oil was measured by the ultraviolet spectrophotometer (UV752, Shunyuhengping instrument Co., LTD, Shanghai, China) at 284 nm after diluting.

### 2.3. Identification of the Bacteria

#### 2.3.1. Morphological Identification

The morphological characterization of the selected isolate was performed by Gram staining and scanning electron microscopy (SEM). 

Gram staining was performed as proposed by Siamak et al. [35]. Cells were stained with crystal violet dye for 1 min and washed away the remaining dye. Iodine solution was added to form a complex between the crystal violet and iodine. Next, ethyl alcohol was added in order to decolorize the cells and carbolic acid reared solution was added to the redye sample. 

SEM was used to visualize the morphology of stain [36]. The strains growing on the inclined plane 2216E were re-suspended with PBS buffer. Cells suspension was centrifuged (6000× *g*, 5 min) and washed with PBS buffer. Cells were fixed in 2.5% glutaraldehyde solution overnight, and then centrifuged (6000× *g*, 5 min) and washed with PBS buffer. cells were dehydrated using a gradient ethanol solution. Afterward, cells were lyophilized and gold-plated. The size and shape of the bacterium were observed using SEM (Supra 55, Carl Zeiss AG, Oberkochen, Germany).

#### 2.3.2. Molecular Identification

The selected strain was identified by 16S rRNA gene sequencing [24], and the sequnce was submitted to the GeneBank of National Center for Biotechnology Information (NCBI) under accession number: OL913102. Sequencing was performed by Lifei biotechnology Co., Ltd. (Shanghai, China). The sequence was entered into the nucleotide basic local alignment search tool (BLASTn) of NCBI to obtain the closely related sequences and estimate the degree of similarity to these nearest relatives [37]. Phylogenetic tree was constructed using the neighbor-joining DNA distance algorithm using software MEGA (version 5) with 1000 bootstrap replictes [38].

### 2.4. Extraction of Biosurfactant

Analysis of surface tension was performed as previously described [33], albeit slightly modified. In order to obtain the produced biosurfactant, experiments were performed in 400 mL of MMC in a conical flask with 1000 mL capacity. Each conical flask was inoculated with 5 mL isolated bacteria (grown 24 h in 2216E liquid medium at 4 °C and 180 rpm to an initial cell concentration of OD_600_ = 1.5). The cultures were supplemented with diesel oil (0.5%, *v*/*v*) and incubated at 4 °C and 180 rpm for 10 days. The fermentation broth was centrifuged at 10,000× *g* for 20 min at 4 °C to obtain the cell-free supernatant. The cell-supernatant was adjusted to pH 2 with addition of 6 M HCl, and stored at 4 °C overnight. After precipitation of the acid, a mixture of chloroform/methanol (2:1 *v*/*v*) was added, the mixture was centrifuged at 8000× *g* for 20 min at 4 °C, and the extracts were finally obtained by rotary evaporation at 40 °C with a rotary evaporator (RE-2000A, Yuhua instrument Co., LTD, Gongyi, China). Purification of the crude extracts was then performed by column chromatography. The crude extracts were dissolved in chloroform and applied to a silica gel (100–120 mesh size). The loaded column was washed with 100 mL chloroform to completely remove the neutral lipids. The mobile phases of different chloroform-methanol ratios were applied to isolate the biosurfactant in sequence: 80:20 *v/v* (100 mL) and 35:65 *v/v* (100 mL) at a flow rate of 1 mL/min. The purified biosurfactant fractions were combined and then dried by rotary evaporation at 40 °C. 

### 2.5. Identification of Biosurfactant

The biosurfactant was identified with TLC and FTIR [12,39]. 

The active component of the surfactant was separated using silica gel plate with a mobile phase of chloroform-methanol-water (65:25:4 *v*/*v*/*v*). To detect the glycolipid-type biosurfactant, the dry plates were sprayed with phenol reagent (3 g phenol in 5 mL sulfuric acid mixed with 95 mL ethanol) and then incubated at 110 °C for 5 min until brown spots appeared [39]. 

The purified surfactant was directly mounted on diamond stub and pressed. The screening of functional groups was performed using a transmission mode scan in spectral region from 400 to 4000 cm^−1^. FTIR spectra were recorded on a Nicolet iS5 Fourier Transform Infrared Spectrometer (Thermo Fisher Scientific, Waltham, USA) [12].

### 2.6. Critical Micelle Concentration (CMC) Measurement

The CMC is the lowest concentration of surfactant molecules associated in a solution to form micelles. The CMC was measured by plotting the surface tension of different concentration of the cell-free supernatant from 0–120 mg/L [33].

### 2.7. Influence of Environmental Factors on Biosurfactant Stability

The effects of temperature, pH, and salinity on the surface activity of the biosurfactant were determined. The analysis was carried out using the cell-free culture supernatant obtained after centrifuging the culture sample at 8000× *g* for 20 min to remove the cells. The pH of the cell-free supernatant was adjusted to 2.0, 3.0, 4.0, 5.0, 6.0, 7.0, 8.0, 9.0, 10.0, 11.0, and 12.0 with addition of 1 N HCl or 1 N NaOH. To assess the effect of temperature on biosurfactant stability, the cell-free supernatant was treated at different temperatures (−18, −2, 0, 10, 25, 35, 55, and 105 °C). Similarly, the sodium chloride concentration of cell-free supernatant was varied from 1–18%. Finally, the stability of the biosurfactant was determined indirectly by measurement of surface tension and emulsification activity (E_24_) [33,40].

Following the method outlined in Teixeira et al. [41], emulsification activity (E_24_) was measured. Cell-free culture supernatant (5 mL) mentioned above was mixed vigorously with diesel oil (5 mL) and followed by incubated for 24 h. E_24_ index value was calculated as percentage of the height of emulsified layer divided by the total height of the liquid column [40]. 

### 2.8. Influence of Carbon Source on Biosurfactant Production 

The isolated cultured bacterial strains (OD_600_ = 1.5) were separately inoculated into 100 mL of MMC with different carbon sources. Diesel (1% *v*/*v*), crude oil (1% *v*/*v*), glucose (10 g/L), sucrose (10 g/L), hexadecane (1% *v*/*v*), phenanthrene (10 g/L), and pyrene (10 g/L) were tested as carbon sources separately. The mixtures were cultured at 4 °C at 180 rpm, and the surface tension and the bacterial concentration were measured after 10 days.

### 2.9. Biodegradation of Crude Oil

The crude oil biodegradation experiments were performed in 50 mL Erlenmeyer flasks containing 25 mL of MMC and 1% crude oil. The medium was sterilized and inoculated with 1% inoculum (optical density of 1.5 at 600 nm) of the petroleum-degrading bacterium. The experiments were conducted under two different conditions, one of which was designed as control group with 25 mL MMC + 1% crude oil and another was composed of 25 mL MMC + 1% isolated bacteria + 1% crude oil. Every group were shaken at speed of 180 rpm at 4 °C. After 21 days, the mixture was extracted with 25 mL of petroleum ether and the organic phase was filtered by anhydrous sodium sulfate to obtain the rest of crude oil. The concentration of petroleum was measured by the ultraviolet spectrophotometer (UV752, Shunyuhengping instrument Co., LTD, Shanghai, China) at 284 nm after diluting [34].

## 3. Results and Discussion

### 3.1. Isolation and Identification of the Petroleum Degrading and Surfactant Producing Bacteria 

Eight petroleum degrading and surfactant producing bacteria were isolated from seawater. Based on diesel oil degradation and surfactant producing abilities, one strain was selected from the eight isolates (Figure S1). 

**Figure 1:**
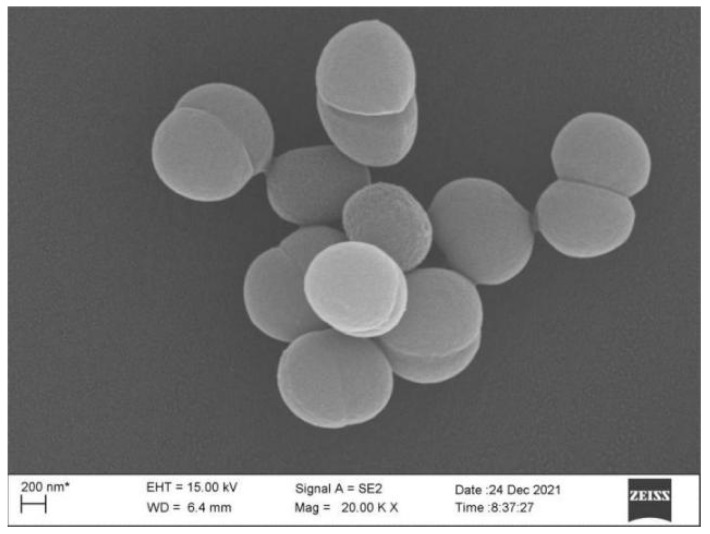
Scanning electron micrographs of cells of *Planococcus* XW-1.

The colony of the strain XW-1 was round, yellow-orange protrusions, the surface was not very smooth. At the end of the Gram stain, the cells were purple, indicating that *Planococcus* sp. XW-1 was Gram positive (Figure S2). The cells of *Planococcus* sp. XW-1 are spherical, and their diameters are about 0.8 µm (Figure 1). 

**Figure 2:**
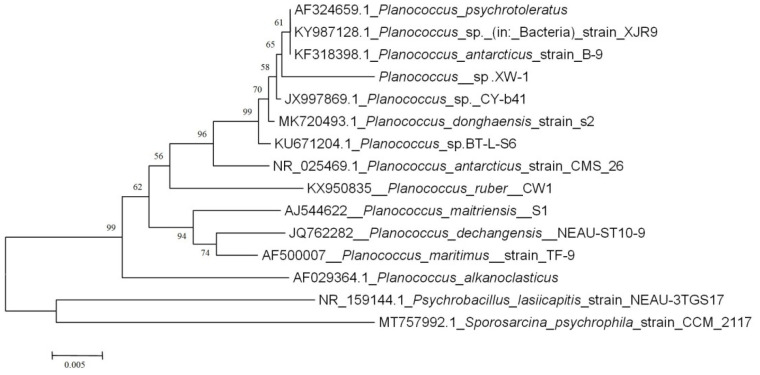
Phylogenetic tree of *Planococcus* sp. XW-1 based on 16S rRNA gene sequences. The phylogenetic tree revealed the relationship between members of the genus *Planococcus* and related genera.

According to the results of BLAST (Table S1), the strain XW-1 showed 98% similarity with *Planococcus*. Phylogenetic tree derived from 16S rRNA sequences was showed in Figure 2. Strain XW-1 appeared to represent a phylogenetically coherent group with *Planococcus psychrotoleratus* and *Planococcus antarcticus*.

*Planococcus* is a typical extreme environmental microorganism, which is often obtained from the deep ocean and sediments, glaciers, frozen soils, Antarctic desert and sea ice salt water, and oil pollution areas [41] such as *Planococcus* donghaensis MPA1U2 isolated from the surface of the Pacific Ocean [42], and *Planococcus* antarcticus DSM 14505 isolated from Antarctic [43]. 

### 3.2. Identification of the Biosurcatant

**Figure 3:**
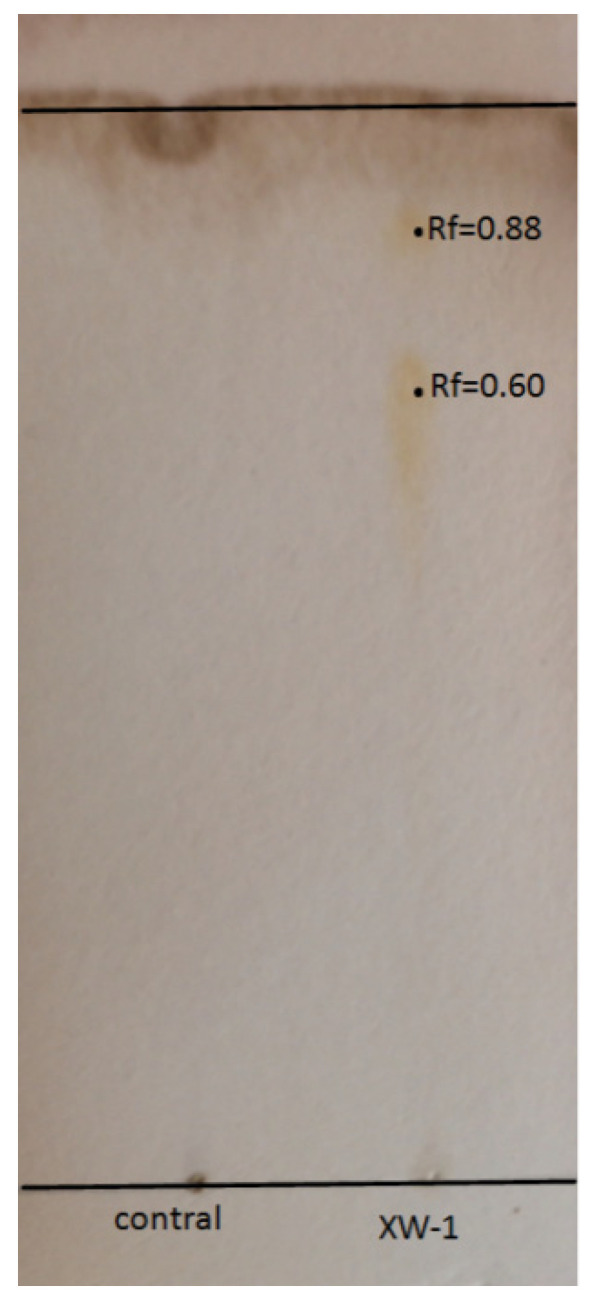
The result of detecting biosurfactant XW-1 by TLC. Without adding biosurfactant were set up as negative control.

The extracted biosurfactant was subjected to TLC analysis to determine its composition. The spot of biosurfactant on plat appeared brown, indicating the presence of glycolipid-type biosurfactant, as shown in Figure 3. Two spots had Rf values of 0.60 and 0.88. The spot with Rf value of 0.6 indicated relation with di-rhamnolipid moieties and the spot with Rf value of 0.88 was consistent with mono-rhamnolipid moieties [44,45]. In a similar study, a mixture of rhamnolipid by *P. aeruginosa* NCIM 5514 yielded spots of Rf 0.57 and 0.89 [46]. 

**Figure 4:**
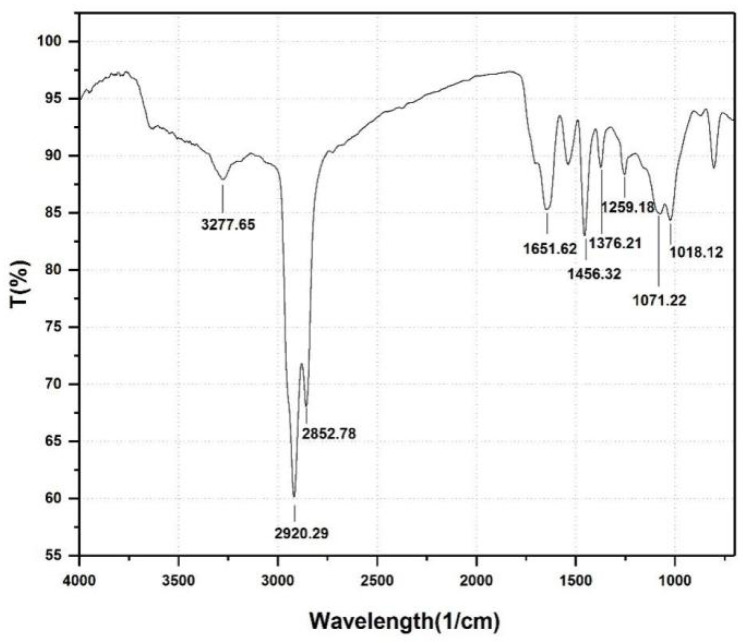
The infrared spectrum of the biosurfactant produced by *Planococcus* sp. XW-1.

As shown in Figure 4, the molecular components of the extract were identified by FTIR. The detected adsorption peaks in wavenumber regions of 3277, 2920, 2852, 1651, and 1300–1000 cm^−1^ indicated the presence of chemical structures that consisted of rhamnose rings and long hydrocarbon chains, similar to rhamnolipids [32]. A strong absorption band near 3277.65 cm^−1^ indicated that the extract included a large amount of -OH. The absorption bands around 2920.29 cm^−1^ and 2852.78 cm^−1^ were caused by the stretching vibration of -CH, -CH2 and -CH3 groups of aliphatic chains [47,48]. Deformation vibration of -CH was observed at 1259.18 cm^−1^ and 1376.21 cm^−1^ [49]. The absorption band near 1456.32 cm^−1^ correlated with ester carbonyl groups and the weak adsorption band around 1651.62 cm^−1^ indicated ester compounds. The stretching vibration of C-O-C bond was observed at 1071.22 cm^−1^ and 1018.12 cm^−1^, suggesting lactone structure and glycoside bond in the molecule [50]. A previous analysis of extract produced by *Pseudomonas aeruginosa* HAK01 determined a structure similar to this structure of XW-1 biosurfactant [50]. These results indicated that the biosurfactant produced by XW-1 has a glycolipid-type biosurfactant structure.

### 3.3. Determination of CMC Value

**Figure 5:**
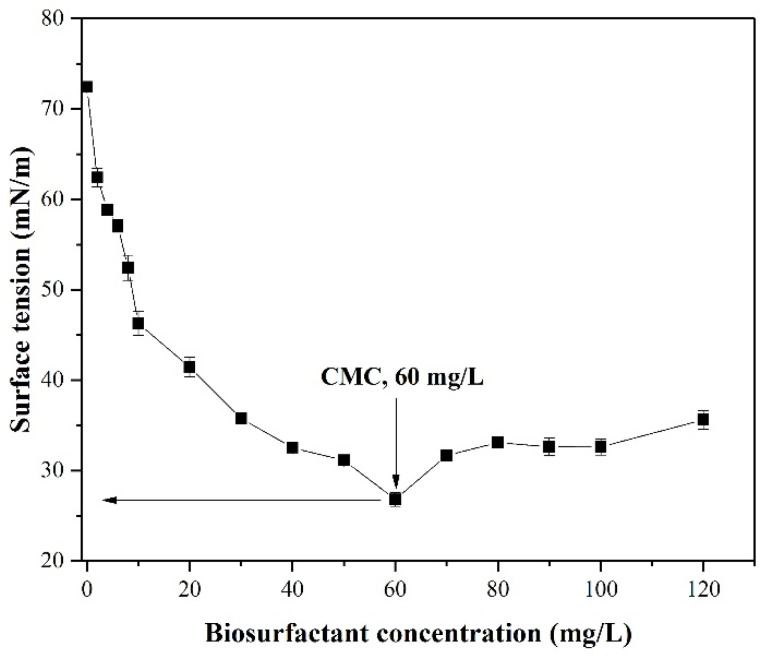
Surface tension versus concentration of isolated biosurfactant produced by *Planococcus* sp. XW-1.

Biosurfactants are highly efficient surfactants that decrease the surface tension of liquid [51,52]. The reduction of surface tension depends on the CMC value, which is obtained by determining the surface tension of different concentrations of biosurfactant solution [32,53]. The biosurfactant value is the concentration required to reach saturated adsorption on the surface. A lower concentration of biosurfactant to form micelles is required with a decrease of CMC. As shown in Figure 5, the surface tension of aqueous solution decreased when concentration of biosurfactant increased to 60 mg/L. At 20 mg/L, surface tension exhibited a significant downward tendency from 72.4 to 41.5 mN/m. Between 20 and 60 mg/L, surface tension decreased moderately, from 41.5 to 26.8 mN/m. More than 60 mg/L, it showed an insignificant ascending tendency along with an increasing biosurfactant concentration, and this phenomenon may be related to the arrangement of surfactant molecules at the gas-liquid interface. Therefore, the concentration of biosurfactant (60 mg/L) was taken as the CMC value. The low CMC value obtained indicates that the biosurfactant produced by XW-1 has excellent formation and aggregation ability.

### 3.4. Effect of Temperature, pH and Salinity on the Stability of Biosurfactant

Environmental factors can affect biosurfactant activity, including temperature, pH, and salinity. Considering the applicability of biosurfactants in marine environment bioremediation, the effectiveness of biosurfactant should be evaluated under conditions of different temperature, pH, and salinity [54]. Surface tension and emulsification activity are important activity parameters of biosurfactants. 

**Figure 6:**
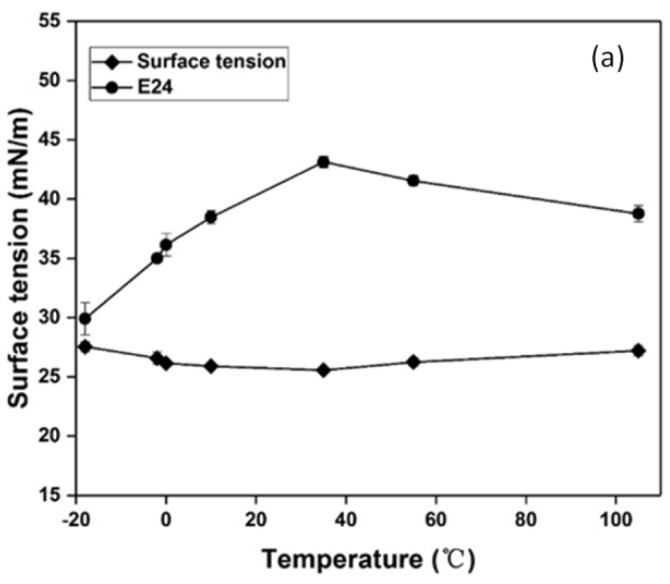

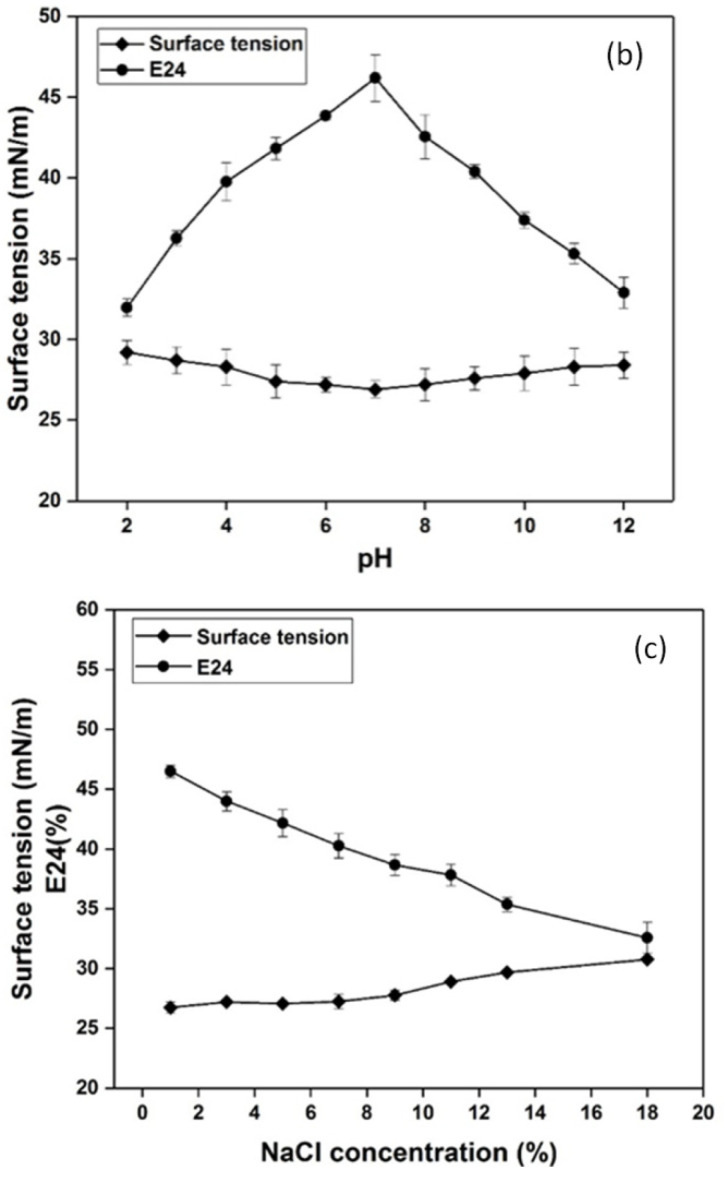
Effect of temperature (**a**), pH (**b**) and NaCl concentration (**c**) on the surface tension and emulsifying property (E_24_) of XW-1 biosurfactant.

The surface tension of the XW-1 biosurfactant was tested from −18 °C to 105 °C. The surface activity was stable at both low and high temperatures, with no significant change even at −18 °C and 105 °C, according to Figure 6a. Surface tension was maintained around 26 mN/m, indicating no loss of activity. Although the emulsification activity was low (only about 30–45%), it was stable at different temperatures. Cooling the supernatant to −18 °C did not cause significant changes in surface activity or emulsification capacity (surface tension was 27.5 mN/m; E_24_ was 29.9%). The biosurfactant produced by XW-1 exhibited an excellent surface activity in extreme environmental conditions, especially at low temperatures. This means that the biosurfactant could be used to enhance bioremediation of petroleum contaminated seawater in the north Yellow Sea in winter.

In similar research, the surface activity of biosurfactant produced by *S. marcescens* maintained stability at temperatures ranging from 30 to 100 °C [55]. According to Aparna et al. [56], the surface activity of the *Pseudomonas* sp. extract isolated from hydrocarbon-contaminated soil samples in Karnataka of India was stable under different temperatures from 4 °C to 121 °C (surface tension was between 34 and 37 mN/m). The emulsification activity of *Planococcus rifietoensis* IITR53 and *Planococcus halotolerans* IITR55 was retained under temperatures from 4 °C to 120 °C [34].

When the pH of the cell-free supernatant was varied from 2 to 12, the surface tension remained relatively stable under neutral and acidic conditions, as shown in Figure 6b. When the pH was adjusted to 2, 3, 4, 5, and 6, the surface tension values were 29.2, 28.7, 28.3, 27.4, and 27.2 mN/m and the emulsification indexes were 32%, 36.3%, 39.8%, 41.8%, and 43.9%, respectively. The surface performance and emulsification capacity of the biosurfactant decreased linearly from pH 4 to 2 since the extreme pH conditions changed the structures of the biosurfactant and altered its surface activity [56]. When the cell-free supernatant was alkalized to pH values of 8, 9, 10, 11, and 12, the surface activities were 27.2, 27.6, 27.9, 28.3, and 28.4 mN/m, respectively, and the emulsification indexes were 44.9%, 42.6%, 40.4%, 38.8%, and 35.7%, respectively. The highest values for surface tension (29.2 mN/m) and the lowest values of emulsification (32%) were obtained at pH 2. The lowest values of surface tension (26.2 mN/m) and the highest values of E_24_ (46.2%) were obtained when the pH value was maintained at 7. Therefore, the biosurfactant was more active under neutral conditions. This result was similar to that reported for rhamnolipid produced by *Pseudomonas* sp. 2B, with surface tension values close to 30 mN/m for various pH values [56]. The pH range of the surface seawater of the Yellow Sea is about 8.10~8.14 [57]. At this range, the biosurfactant produced by *Planococcus* sp. XW-1 had well surface activities. Figure 6c shows the stability of biosurfactant in different concentrations of NaCl. The surface tension was stable at 27 mN/m from 1–9% (*w*/*v*) NaCl, indicated that the surfactant could be used for a variety of marine environments, such as salt marshes, estuaries, intertidal zones. At higher concentration, surface tension increased to 30.8 mN/m and the emulsification index decreased from 46.5% to 32.6% at 18% NaCl concentration. This effect at high salt is due to the reduction in the micelles size and shape at high salt concentrations, affecting the functional properties of the biosurfactant [58]. Electrolytes had a direct effect on the carboxylate groups of the rhamnolipids. With strong repulsive electrostatic forces between the rhamnolipid molecules, the solution/air interface had a net negative charge. This negative charge was shielded by the Na^+^ ions in the electrical double layer in the presence of NaCl, causing the formation of a close-packed monolayer and a decrease in surface tension values [56,59]. This observed effect of salinity on biosurfactant was consistent with previous reports [60,61,62].

### 3.5. Effect of Seven Carbon Sources on Biosurfactant Production

**Figure 7:**
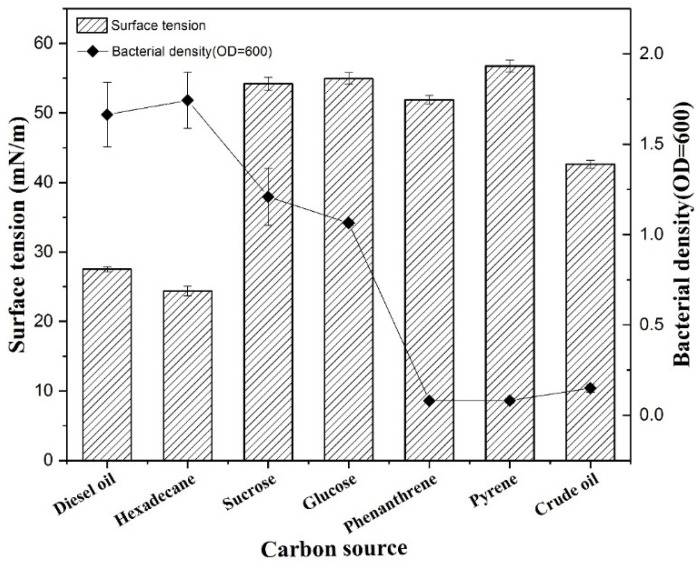
Effect of different carbon sources on surface tension and bacterial density.

The influence of different carbon sources (diesel oil, hexadecane, sucrose, glucose, phenanthrene, pyrene, and crude oil) on biosurfactant production was characterized and is shown in Figure 7. Obvious differences were observed by testing the effects of these seven carbon sources on biosurfactant production. The surface tension value decreased from 70.1 to 24.6 mN/m when hexadecane was used as the sole carbon source, and decreased from 70.1 to 26.8 mN/m when diesel oil was used as the only carbon source. Therefore, the production of the XW-1 biosurfactant using either hexadecane or diesel oil as the sole carbon source resulted in higher surface activity. 

As shown in Figure 7, the growth rate of the cells was higher when hexadecane or diesel oil was used as the sole carbon source. Compared with phenanthrene, pyrene, and crude oil, the surface tension of the fermentation liquid changed little but there was higher growth of cells using sucrose and glucose as carbon sources. Glucose and sucrose are good carbon sources for microbial growth due to their high bioavailability, but the synthesis of secondary metabolites of microorganisms is often repressed by catabolites, such as gluconic acid (produced by glucose and sucrose), which is a common phenomenon [32]. The surface tension value of the fermentation showed little change with phenanthrene and pyrene as carbon sources. Compared to production in the presence of phenanthrene and pyrene, the biosurfactant produced by XW-1 when crude oil was used as a carbon source showed higher surface activity (surface tension was 43 mN/m). However, the growth rates of these cells were lowest due to low bioavailability. The addition of biosurfactants can promote degradation for the removal of phenanthrene, pyrene, and crude oil. 

### 3.6. Biodegradation of Crude Oil

**Figure 8:**
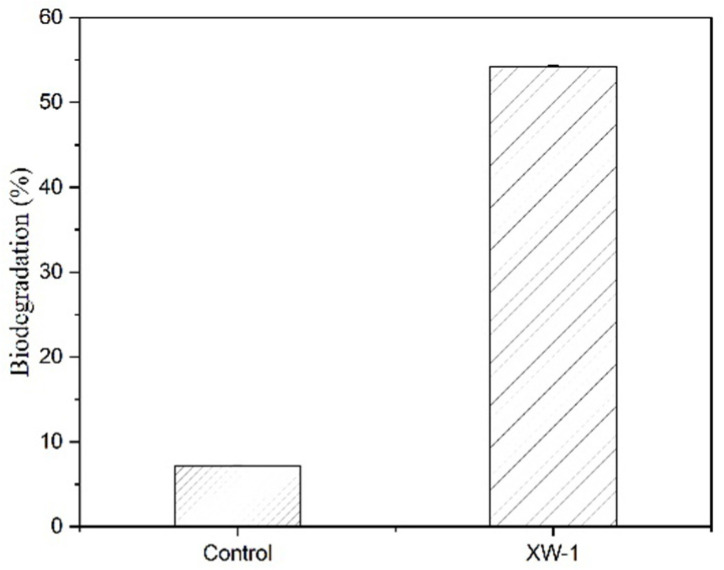
Biodegradation rate of crude oil at 4 °C for 21 days (samples without adding bacteria were set up as the control).

After 21 days of growth at 4 °C in medium with 1% crude oil and 1% bacteria broth, 54% of crude oil was degraded (Figure 8). The result showed that the strain XW-1 has a good ability to degrade petroleum at low temperature.

According to Hou [63], the average concentrations of dissolved petroleum hydrocarbons in the surface water of north Yellow Sea in winter was about 0.051 mg/l, which slightly beyond the concentration limit of Grade I(II) seawater according to National Seawater Standard, China. Moreover, the petroleum hydrocarbon concentration in the north Yellow Sea is higher in the west and lower in the east in winter. So, decontamination of petroleum is therefore necessary on the west coast of the north Yellow Sea. There are some bacteria can degrade various hydrocarbons and therefore can contribute significantly to remove hydrocarbon pollutants in marine environments [13,14]. For example, *Planococcus* ZD22 can fully degraded 2 mM of benzene at 8 °C for five days [64], and *Rhodococcus*.sp stain Q15 degraded almost all of the n-alkanes after 28 days of growth at 0 °C in MSM containing 0.1% diesel oil [65].

## 4. Conclusions

*Planococcus* sp. XW-1, isolated from the west coast of the north Yellow Sea, showed outstanding abilities in petroleum degrading and surfactant producing at low temperatures. Up to 54% of crude oil was degraded by adding *Planococcus* sp. XW-1 at 4 °C. The biosurfactant produced by *Planococcus* sp. XW-1 was glycolipid-type biosurfactant, and the CMC was 60 mg/L. The biosurfactant was able to reduce the surface tension of water to 26.8 mN/m. Meanwhile, it exhibited a robust tolerance for temperature, pH, and salinity. These findings suggest that *Planococcus* sp. XW-1 is a promising candidate for use in *in-situ* bioremediation of petroleum-contaminated marine ecosystems of the north Yellow Sea in winter. 

## Data Availability

Data is in agreement with the MDPI Research Data Policies.

## Supplementary Materials

The following supporting information can be downloaded at: https://www.mdpi.com/article/10.3390/ijerph19020782/s1, Figure S1. The abilities of isolated strains in biosurfactant production and diesel oil degradiation; Figure S2: Gram staining of Plancoccus sp.XW-1; Table S1. The highly similar sequence with stain XW-1 based on the BLAST result

## References

[1] Gargouri B., Karray F., Mhiri N., Aloui F., Sayadi S. (2014). Bioremediation of petroleum hydrocarbons-contaminated soil by bacterial consortium isolated from an industrial wastewater treatment plant. J. Chem. Technol. Biotechnol..

[2] Xia W., Du Z., Cui Q., Dong H., Wang F., He P., Tang Y. (2014). Biosurfactant produced by novel *Pseudomonas* sp. WJ6 with biodegradation of n-alkanes and polycyclic aromatic hydrocarbons. J. Hazard. Mater..

[3] Chandankere R., Yao J., Cai M., Masakoralaabe K., Jain A.K., Martin M.F.C. (2014). Properties and characterization of biosurfactant in crude oil biodegradation by bacterium *Bacillus methylotrophicus* USTBa. Fuel.

[4] Mohn W.W., Stewart G.R. (2000). Limiting factors for hydrocarbon biodegradation at low temperature in Arctic soils. Soil Biol. Biochem..

[5] Borah D., Yadav R.N.S. (2016). Bioremediation of petroleum based contaminants with biosurfactant produced by a newly isolated petroleum oil degrading bacterial strain. Egypt. J. Pet..

[6] Giudice A.L., Fani R. (2015). Cold-adapted bacteria from a coastal area of the Ross Sea (Terra Nova Bay, Antarctica): Linking microbial ecology to biotechnology. Hydrobiologia.

[7] Fingas M.F. (2004). Modeling evaporation using models that are not boundary-layer regulated. J. Hazard. Mater..

[8] Michaud L., Giudice A.L., Saitta M., De Domenico M., Bruni V. (2004). The biodegradation efficiency on diesel oil by two psychotropic Antarctic marine bacteria during a two-month-long experiment. Mar. Pollut. Bull..

[9] Bezza F.A., Beukes M., Chirwa E.M.N. (2015). Application of biosurfactant produced by *Ochrobactrum intermedium* CN3 for enhancing petroleum sludge bioremediation. Process Biochem..

[10] Petrikov K., Delegan Y., Surin A., Ponamoreva O., Puntus I., Filonov A., Boronin A. (2013). Glycolipids of *Pseudomonas* and *Rhodococcus* oil-degrading bacteria used in bioremediation preparations: Formation and structure. Process Biochem..

[11] Jung J.H., Joe M.H., Kim D.H., Park H., Choi J.I., Lim S.Y. (2018). Complete genome sequence of *Planococcus* sp. PAMC21323 isolated from Antarctica and its metabolic potential to detoxify pollutants. Stand. Genomic. Sci..

[12] Waghmode S., Suryavanshi M., Dama L., Kansara S., Ghattargi V., Das P., Banpurkar A., Satpute S.K. (2019). Genomic Insights of Halophilic Planococcus maritimus SAMP MCC 3013 and Detail Investigation of Its Biosurfactant Production. Front. Microb..

[13] Waghmode S., Suryavanshi M., Sharma D., Satpute S.K. (2020). *Planococcus Species*—An Imminent Resource to Explore Biosurfactant and Bioactive Metabolites for Industrial Applications. Front. Bioeng. Biotechnol..

[14] Kumar A.S., Mody K., Jha B. (2007). Evaluation of biosurfactant/bioemulsifier production by a marine bacterium. Bull. Environ. Contam. Toxicol..

[15] Ebrahimipour G., Gilavand F., Karkhane M., Kavyanifard A., Teymouri M., Marzban A. (2014). Bioemulsification activity assessment of an indigenous strain of halotolerant *Planococcus* and partial characterization of produced biosurfactants. Int. J. Environ. Sci. Technol..

[16] Marchant R., Banat I.M. (2012). Biosurfactants: A sustainable replacement for chemical surfactants. Biotechnol. Lett..

[17] Varjani S.J., Upasani V.N. (2017). Critical review on biosurfactant analysis, purification and characterization using rhamnolipid as a model biosurfactant. Bioresour. Technol..

[18] Ohadi M., Dehghannoudeh G., Forootanfar H., Shakibaie M., Rajaee M. (2018). Investigation of the structural, physicochemical properties, and aggregation behavior of lipopeptide biosurfactant produced by *Acinetobacter junii* B6. Int. J. Biol. Macromol..

[19] Mulligan C.N. (2009). Recent advances in the environmental applications of biosurfactants. Curr. Opin. Colloid Interface Sci..

[20] Pepi M., Cesaro A., Liut G., Baldi F. (2005). An antractic psychrotrophic bacterium *Halomonas* sp. ANT-3b, growing on n-hexadecane, produces a new emulsifying glycolipid. FEMS Microbiol. Ecol..

[21] Bej A.K., Saul D., Aislabie J. (2000). Cold-tolerant alkane-degrading *Rhodococcus* species from Antarctica. Polar Biol..

[22] Ruberto L.A.M., Vazquez S., Lobalbo A. (2005). Psychrotolerant hydrocarbon-degrading *Rhodococcus* strains isolated from polluted Antarctic soils. Antarct. Sci..

[23] Giudice A.L., Casella P., Caruso C., Mangano S., Bruni V., Domenico M.D., Michaud L. (2010). Occurrence and characterization of psychrotolerant hydrocarbon-oxidizing bacteria from surface seawater along the Victoria Land coast (Antarctica). Polar Biol..

[24] Aislabie J., Foght J., Saul D. (2000). Aromatic hydrocarbon-degrading bacteria from soil near Scott Base, Antarctica. Polar Biol..

[25] Farrell R.L., Rhodes P.L., Aislabie J. (2003). Toluene-degrading Antarctic Pseudomonas strains from fuel-contaminated soil. Biochem. Biophys. Res. Commun..

[26] Saul D.J., Aislabie J.M., Brown C.E., Harris L., Foght J.M. (2005). Hydrocarbon contamination changes the bacterial diversity of soil from around Scott Base, Antarctica. FEMS Microbiol. Ecol..

[27] Bao X.W., Wang X.Q., Gao G.P., Wu D.X. (2002). The characteristics of the seasonal variability of the sea surface temperature field in the Bohai Sea, the Huanghai Sea and the East China Sea from AVHRR data. Acta Oceanol. Sin..

[28] Wang C., He S., Li Y. (2009). Research on the status of marine oil spill pollution in China and its ecological impact. Mar. Sci..

[29] Li L., Shen X., Zhao C., Liu Q., Liu X., Wu Y. (2019). Biodegradation of dibenzothiophene by efficient *Pseudomonas* sp. LKY-5 with the production of a biosurfactant. Ecotoxicol. Environ. Saf..

[30] Cheng X.Y., Liu W.W., Xu Y., Zhou N.Y. (2019). Screening and characterization of culturable hydrocarbon-degrading strains from the South and East China Seas. J. Tianjin Univ. Technol..

[31] Hassanshahian M., Emtiazi G., Cappello S. (2012). Isolation and characterization of crude-oil-degrading bacteria from the Persian Gulf and the Caspian Sea. Mar. Pollut. Bull..

[32] Pornsunthorntawee O., Wongpanit P., Chavadej S. (2008). Structural and physicochemical characterization of crude biosurfactant produced by *Pseudomonas aeruginosa* SP4 isolated from petroleum-contaminated soil. Bioresour. Technol..

[33] Durval I.J.B., Mendonça A.H.R., Rocha I.V., Luna J.M., Rufino R.D., Converti A., Sarubbo L.A. (2020). Production, characterization, evaluation and toxicity assessment of a *Bacillus cereus* UCP 1615 biosurfactant for marine oil spills bioremediation. Mar. Pollut. Bull..

[34] Xue J.L., Wu Y.N., Liu Z.X., Li M.L., Sun X.Y., Wang H.J., Liu B. (2017). Characteristic Assessment of Diesel-degrading Bacteria Immobilized on Natural Organic Carriers in Marine Environment: The Degradation Activity and Nutrient. Sci. Rep..

[35] Siamak P.Y., Henning S., Hans J.S.L., Geir G. (2001). Use of magnetic beads for Gram staining of bacteria in aqueous suspension. J. Microbiol. Methods.

[36] Atakpa E.O., Zhou H.H., Jiang L.J., Ma Y.H., Liang Y.P., Li Y.H., Zhang D.D., Zhang C.F. (2022). Improved degradation of petroleum hydrocarbons by co-culture of fungi and biosurfactant-producing bacteria. Chemosphere.

[37] Tamura K., Peterson D., Peterson N., Stecher G., Nei M., Kumar S. (2011). MEGA5: Molecular evolutionary genetics analysis using maximum likelihood, evolutionary distance, and maximum parsimony methods. Mol. Biol. Evol..

[38] Altschul S.F., Gish W., Miller W., Meyers E.W., Lipman D.J. (1990). Basic local alighnment search tool. J. Mol. Biol..

[39] Gaur V., Tripathi V., Gupta P., Dhiman N., Regar R.K., Gautam K., Srivastava J.K., Patnaik S., Patel D.K., Manickam N. (2020). Rhamnolipids from *planococcus* spp. and their mechanism of action against pathogenic bacteria. Bioresour. Technol..

[40] Cooper D.G., Goldenberg B.G. (1987). Surface-active agents from two *Bacillus* species. Appl. Environ. Microbiol..

[41] Teixeira S.K.S., Gudiña E.J., Schwan R.F., Rodrigues L.R., Dias D.R., Teixeira J.A. (2018). Improvement of biosurfactant production by *Wickerhamomyces anomalus* CCMA 0358 and its potential application in bioremediation. J. Hazard. Mater..

[42] Pearson M.D., Noller H.F. (2011). The draft genome of *Planococcus donghaensis* MPA1U2 reveals nonsporulation pathways controlled by a conserved Spo0A regulon. J. Bacteriol..

[43] Abelardo M., Miguel G., Borja S. (2012). Genome sequence of the Antarctic psychrophile bacterium *Planococcus antarcticus* DSM 14505. J. Bacteriol..

[44] Lotfabad T.B., Abassi H., Ahmadkhaniha R., Roostaazad R., Masoomi F., Zahiri H.S., Ahmadian G., Vali H., Noghabi K.A. (2010). Structural characterization of a rhamnolipid-type biosurfactant produced by *Pseudomonas aeruginosa* MR01: Enhancement of di-rhamnolipid proportion using gamma irradiation. Colloids Surf. B Biointerfaces.

[45] Raza Z.A., Khalid Z.M., Banat I.M. (2009). Characterization of rhamnolipids produced by a *Pseudomonas aeruginosa mutant* strain grown on waste oils. J. Environ. Sci. Health Part A.

[46] Varjani S.J., Upasani V.N. (2016). Core Flood study for Enhanced Oil Recovery through ex-situ bioaugmentation with thermo-and halo-tolerant rhamnolipid produced by *Pseudomonas aeruginosa* NCIM 5514. Bioresour. Technol..

[47] Abouseoud M., Maachi R., Amrane A., Boudergua S. (2008). Evaluation of different carbon and nitrogen sources in production of biosurfactant by *Pseudomonas fluorescens*. Desalination.

[48] Saikia R.R., Deka S., Deka M., Banat I.M. (2012). Isolation of biosurfactant-producing *Pseudomonas aeruginosa* RS29 from oil-contaminated soil and evaluation of different nitrogen sources in biosurfactant production. Ann. Microbiol..

[49] Li S., Pi Y., Bao M., Zhang C., Zhao D., Li Y., Sun P., Lu J. (2015). Effect of rhamnolipid biosurfactant on solubilization of polycyclic aromatic hydrocarbons. Mar. Pollut. Bull..

[50] Khadenolhosseini R., Jafari A., Mousavi S.M., Hajfarajollah H., Noghabi K.A., Manteghian M. (2019). Physicochemical characterization and optimization of glycolipid biosurfactant production by a native strain of *Pseudomonas aeruginosa* HAK01 and its performance evaluation for the MEOR process. RSC Adv..

[51] Bezza F.A., Chirwa E.M.N. (2017). The Role of Lipopeptide Biosurfactant on Microbial Remediation of Aged Polycyclic Aromatic Hydrocarbon (PAHs)-contaminated Soil. Chem. Eng. J..

[52] Parkinson M. (1985). Bio-surfactants. Biotechnol. Adv..

[53] Kaustuvmani P., Rupshikha P., Kalita M.C., Deka S. (2017). Characterization of Biosurfactant Produced during Degradation of Hydrocarbons Using Crude Oil As Sole Source of Carbon. Front. Microbiol..

[54] Heryani H., Putra M.D. (2017). Kinetic study and modeling of biosurfactant production using, *Bacillus* sp.. Electron. J. Biotechnol..

[55] Almansoory A.F., Hasan H.A., Abdullah S.R.H., Idris M., Anuar N., AL-Adiwish W.M. (2019). Biosurfactant produced by the hydrocarbon-degrading bacteria: Characterization, activity and applications in removing TPH from contaminated soil. Environ. Technol. Innov..

[56] Aparna A., Srinikethan G., Smitha H. (2012). Production and characterization of biosurfactant produced by a novel *Pseudomonas* sp. 2B. Colloids Surf. B Biointerfaces.

[57] Shi Q. (2017). Temporal and spatial modes and mechanisms of seasonal cycles of pH fields in the Yellow Sea. J. Appl. Ocean.

[58] De Franca I.W.L., Lima A.P., Lemos J.A.M., Lemos C.G.F., Melo V.M.M., De Santana H.B., Goncalves L.R.B. (2015). Production of a biosurfactant by *Bacillus subtilis* ICA56 aiming bioremediation of impacted soils. Catal. Today.

[59] Helvaci S.S., Peker S., Ozdemir G. (2004). Effect of electrolytes on the surface behavior of rhamnolipids R1 and R2. Colloids Surf. B Biointerfaces.

[60] Dubey K.V., Charde P.N., Meshram S.U., Shendre L.P., Dubey V.S., Juwarkar A.A. (2012). Surface-active potential of biosurfactants produced in curd whey by *Pseudomonas aeruginosa* strain-PP2 and Kocuria turfanesis strain-J at extreme environmental conditions. Bioresour. Technol..

[61] Khopade A., Biao R., Liu X., Mahadik K., Zhang L., Kokare C. (2012). Production and stability studies of the biosurfactant isolated from marine *Nocardiopsis* sp. B4. Desalination.

[62] Anyanwu C.U., Obi S.K.C., Okolo B.N. (2011). Lipopeptide biosurfactant production by *Serratia marcescens* NSK-1 strain isolated from petroleum-contaminated soil. J. Appl. Sci. Res..

[63] Hou J.N., Zhang C.S., Shi X.Y. (2011). Seasonal variations and distribution characteristics of petroleum hydrocarbons in northern Yellow Sea. Prog. Fish. Sci..

[64] Li H., Liu Y.H., Luo N., Zhang X.Y., Luan T.G., Hu J.M., Wang Z.Y., Wu P.C., Chen M.J., Lu J.Q. (2006). Biodegradation of benzene and its derivatives by a psychrotolerant and moderately haloalkaliphilic *Planococcus* sp. strain ZD22. Res. Microbiol..

[65] Whyte L.G., Hawari J., Zhou E., Bourbonnière L., Inniss W.E., Greer C.W. (1998). Biodegradation of variable-chain-length alkanes at low temperatures by apsychrotrophic *Rhodococcus* sp.. Appl. Environ. Microbiol..

