# Supplementary material for: Isolation and Characterization of a Biosurfactant Producing Strain Planococcus sp. XW-1 from the Cold Marine Environment
Source: Int J Environ Res Public Health. 2022 Jan 11;19(2):782. doi: 10.3390/ijerph19020782 (PMC8776024; doi:10.3390/ijerph19020782)
Supplement: Supplementary file 1 [file ijerph-19-00782-s001.zip › ijerph-1496809-supplementary.pdf]

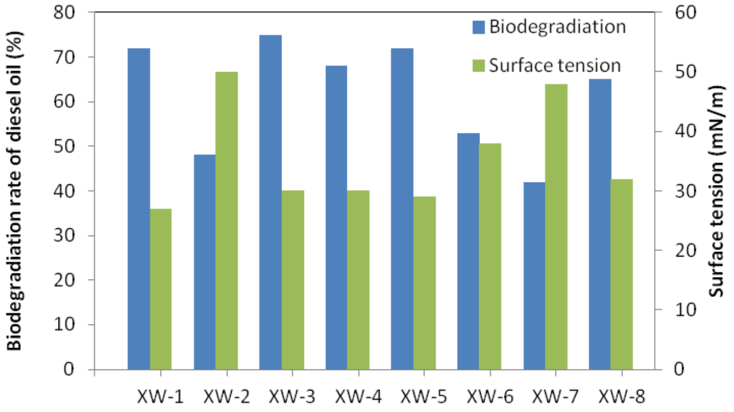

Figure S1. The abilities of isolated strains in biosurfactant production and diesel oil degradation

Table S1. The highly similar sequence with stain XW-1 based on the BLAST result

Strain name	Query cover	Per ident	Accession
Plancoccus donghaensis strain S2	99%	97.98%	MK720493.1
Plancoccus antarcticus strain B-9	98%	97.97%	KF318398.1
Plancoccus psychrotoleratus strain B-9	98%	97.97%	AF324659.1
Plancoccus sp.(in:Bacteria)strain XJR9	98%	98.05%	KY987128.1

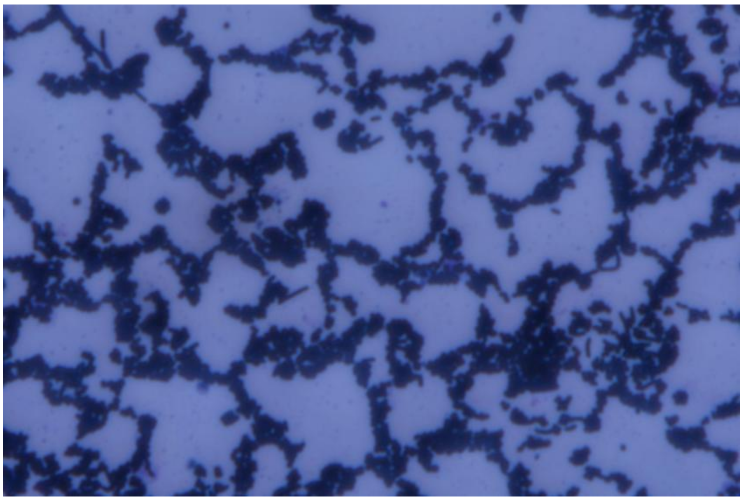

Figure S2. Gram staining of Plancoccus sp.XW-1
